# Understanding determinants of socioeconomic inequality in mental health in Iran's capital, Tehran: a concentration index decomposition approach

**DOI:** 10.1186/1475-9276-11-18

**Published:** 2012-03-26

**Authors:** Esmaeil Khedmati Morasae, Ameneh Setareh Forouzan, Reza Majdzadeh, Mohsen Asadi-Lari, Ahmad Ali Noorbala, Ahmad Reza Hosseinpoor

**Affiliations:** 1 Qom University of Medical Sciences & Health Services, Qom, Iran; 2 Center for Research on Social Determinants of Health, University of Social Welfare and Rehabilitation Sciences, Tehran, Iran; 3 Knowledge Utilization Research Center & Department of Epidemiology and Biostatistics, Public Health School, Tehran University of Medical Sciences, Tehran, Iran; 4 Department of Epidemiology and Biostatistics, Public Health School, Tehran University of Medical Sciences, Tehran, Iran; 5 Roozbeh Psychiatric Hospital, Tehran University of Medical Sciences, Tehran, Iran; 6 Department of Health Statistics and Informatics, Innovation, Information, Evidence and Research Cluster, World Health Organization, Geneva, Switzerland

**Keywords:** Mental health, Socioeconomic inequality, Concentration index, Decomposition, Tehran

## Abstract

**Background:**

Mental health is of special importance regarding socioeconomic inequalities in health. On the one hand, mental health status mediates the relationship between economic inequality and health; on the other hand, mental health as an "end state" is affected by social factors and socioeconomic inequality. In spite of this, in examining socioeconomic inequalities in health, mental health has attracted less attention than physical health. As a first attempt in Iran, the objectives of this paper were to measure socioeconomic inequality in mental health, and then to untangle and quantify the contributions of potential determinants of mental health to the measured socioeconomic inequality.

**Methods:**

In a cross-sectional observational study, mental health data were taken from an Urban Health Equity Assessment and Response Tool (Urban HEART) survey, conducted on 22 300 Tehran households in 2007 and covering people aged 15 and above. Principal component analysis was used to measure the economic status of households. As a measure of socioeconomic inequality, a concentration index of mental health was applied and decomposed into its determinants.

**Results:**

The overall concentration index of mental health in Tehran was -0.0673 (95% CI = -0.070 - -0.057). Decomposition of the concentration index revealed that economic status made the largest contribution (44.7%) to socioeconomic inequality in mental health. Educational status (13.4%), age group (13.1%), district of residence (12.5%) and employment status (6.5%) also proved further important contributors to the inequality.

**Conclusions:**

Socioeconomic inequalities exist in mental health status in Iran's capital, Tehran. Since the root of this avoidable inequality is in sectors outside the health system, a holistic mental health policy approach which includes social and economic determinants should be adopted to redress the inequitable distribution of mental health.

## Background

Mental health is an integral and essential component of health; undeniably, there can be no health without mental health [1,2]. Mental health influences a large range of qualities for individuals and communities, including higher quality of life, better physical health, productivity, social cohesion, and wellbeing [1]. However, mental health is unequally distributed in our societies, meaning that people who live in a socially and economically disadvantaged situation suffer from a disproportionate burden of mental disorders and subsequent adverse consequences [2,3].

The etiology of mental disorders is multifactorial; risk is determined by a combination and interaction of biological, psychological, and social determinants [2]. Social determinants have been shown to account for a remarkably large part of the prevalence and unequal distribution of mental disorders within and among countries [2]. In fact, a number of studies have found convincing evidence implicating socioeconomic position, gender inequality, education, income inequality, racial and ethnical discrimination, and other social factors as determinants of mental disorders [2-4]. However, evidence from low- and middle-income countries has been scarce in this respect and there is a need for more contextual research to enrich the current knowledge and policy pertaining to socioeconomic inequalities in mental health [2].

The Islamic republic of Iran is a developing, middle-income country in the Middle East, where mental health has always had a specific place in the health agenda [4,5]. Since the late 1980s, Iran has sought full integration of mental health into its Primary Health Care (PHC) [5,6]. This integration has helped bring about great improvements in the accessibility of affordable and acceptable mental health care on a national scale and is currently one of the most successful models in the world [6]. However, owing to lack of strong PHC in urban areas, the integration has been much more limited in urban areas compared with rural areas [6,7]. Program coverage was reported to be 21.7% in urban areas and 82.8% in rural areas in 2004 [6].

Moreover, mental disorders have been among the top disabling disorders in Iran; next to unintentional accidents, they rank second in the list of burden of diseases in the country [8]. Recent studies have shown that increasingly more Iranian people are suffering from mental disorders; according to a national survey in 2001, the prevalence of mental disorders was estimated to be around 22% and was worse among women [9].

In terms of inequalities in mental health, limited informative and beneficial data is available in Iran, as with most other developing countries. To be specific, some descriptive studies have revealed that some groups like women, the elderly, unemployed, divorced, widowed, and people of lower education status had higher rates of mental disorders in Iran [9]; however, the magnitude of these inequalities in mental health have not been investigated in detail thus far. Therefore, Iran's mental health system lacks such useful evidence. However, this matter has had its implications for Iranian mental health care and mental health equity has been mostly absent from any proposed health agendas.

Appropriate evidence on health distribution and level is vital for understanding the scale of the problem, assessing the effects of action and monitoring progress [10]. Consequently, it is essential to have a clear picture of the mental health inequalities and their determinants in place and introduce mechanisms to ensure that the data is understood and applied to develop more effective policies, systems, and programs.

However, in order to provide the necessary evidence, Iran has recently taken a great leap to assess the social determinants of health in its megacities by applying the Urban Health Equity Assessment and Response Tool (Urban HEART), a survey piloted and conducted in Iran's capital, Tehran [11]. Fortunately, mental health has been a significant part of Tehran's Urban HEART, in which mental health social determinants-related data is collected. From this, an unprecedented opportunity is being provided to assess mental health inequalities in Iran.

There are different measures for assessing health inequalities, and one common measure is the Concentration Index (CI) [12-14]. This measure can be decomposed into its determinants so that the contribution of these determinants to the inequality could be untangled and quantified [14-18].

Thus, using Tehran's Urban HEART data on mental health and the CI method, the present study, for the first time, aimed to measure socioeconomic mental health inequality in Iran and then decompose the measured inequality into its determinants. Through such analysis and revealing the contribution of each determinant to mental health inequality, we hope to more specifically identify interventional policies and also vulnerable target groups in order to reduce this inequality.

## Methods

### Data

The data for this study were taken from the Urban Health Equity Assessment and Response Tool (Urban HEART) survey, conducted in Tehran in 2007, covering people aged 15 and above. The sampling design was stratified multistage cluster sampling and, to avoid internal cluster correlations, stratified two-dimensional systematic sampling was used [11]. First, Tehran's 22 districts were defined as strata. Then, applying cluster sampling, 120 blocks were chosen from each stratum. In each block, eight households were selected by systematic sampling, meaning that 960 households were chosen from each stratum. The original sample comprised 25,485 people. The response rate in Urban HEART was approximately 87%. Therefore, data from 22,135 people from the original sample were entered into the analyses. Interviewers gathered information on two persons from each household: the head of the household for economic status and a chosen member for mental health data. A self-administered 28-item General Health Questionnaire (GHQ-28) was used as a screening tool for detection of possible cases of mental disorders [19]. The validity and reliability of this questionnaire has been confirmed for Iranians [20]. The best cut-off point, determined by the Likert scoring method, was 24 out of 84, so that those who scored 24 or above were designated as possible cases of mental disorder. Therefore, a binary dependent variable was selected. In this study, the included independent variables were the following: age, gender, marital status, ethnicity, education status, occupation status, household size, health insurance status, district of residence and index of household economic status.

Applying principal components analysis (PCA), we constructed an index of household economic status [21,22]. The asset variables used in the PCA analysis were the following: having a personal computer, freezer, motorcycle, mobile phone, car, kitchen, bathroom, landline, toilet, type of house ownership, residence area per capita and number of rooms per capita. As there were slight and condonable missing data on asset variables, the effect of missing data was not an issue in the construction of the economic status index. After determining economic status, economic quintiles were calculated and used in the subsequent modelling.

### Methodology

#### Concentration index

To measure socioeconomic inequality in mental health, we used a CI approach [12-14]. The CI is defined on the basis of a Concentration Curve (CC) [14]. The CC plots the cumulative percentage of a health variable against the cumulative percentage of population, ranked by economic index from poorest to richest [14]. Two variables are included in a CC: a health variable, and an economic status variable against which the distribution of the health variable is to be examined. If everyone enjoyed the same level of health, regardless of economic status, the CC would be a 45-degree line called the "line of equality." In contrast, if the health variable has higher (lower) values among poorer people, the CC would lie over (under) the line of equality. The farther the curve is away from the equality line, the more unequal is the distribution of the health variable [14]. The CI is defined as twice the area between the CC and the line of equality [23]. Thus, when CC coincides with the equality line, the CI equals zero; however, when the CC is above the equality line, the CI takes a negative value and a positive value when it is below the equality line. The CI is bound between -1 and +1 [14,24].

The CI summarizes the CC and quantifies the degree of economic-related inequality in a health variable [12-14]. Broadly speaking, the CI shows the relationship between health and economic status; its sign indicates the direction of the relationship and its magnitude echoes both the strength of the relationship and degree of variability in the distribution of the health variable [14].

Following Kakwani, the CI can be computed as twice the covariance of the health variable and a person's rank in terms of economic status, divided by the mean of the health variable [12,13]:

(1) $$C=\frac{2}{\mu }\text{cov}\left(y_{i},R_{i}\right)$$

Where y_i _and R_i _are respectively the health status of the ith individual and the fractional rank of the ith individual (in terms of the index of household economic status); μ is the mean of the health and cov denotes the covariance.

### Decomposition of the CI

Following Wagstaff et al [15], we assume that we have a linear regression model linking our health variable of interest y to a set of k determinants (X_k_):

(2) $$y_{i}=\alpha +\sum_{k}{\beta_{k}x_{ki}+\varepsilon }_{i}$$

Where i means ith individual, β_k _denotes the coefficients and ε_i _is an error term (interpersonal variations in y are thus assumed to derive from systematic variations across socioeconomic groups in the determinants of y, i.e. the X_k_).

Given the relationship between y_i _and X_ki _in Equation (2), the CI for y can be written as:

(3) $$C=\sum_{k}\left(\frac{\beta_{k}{\bar{x}}_{k}}{\mu }\right)C_{k}+\frac{GC_{\varepsilon }}{\mu }=C_{Ŷ}+\frac{GC_{\varepsilon }}{\mu }$$

Where μ is the mean of y, ${\bar{X}}_{k}$ is the mean of X_k_, C_k _is the CI for X_k _(defined exactly like C) and in the last term GC_ε _(residual) is the generalized CI for ε_i_.

Equation (3) is clearly made up of two components: (1) a deterministic or explained component and (2) an unexplained component. The first component consists of two constituents: elasticity and a CI of k regressors (determinants). Elasticity $\left(\frac{\beta_{k}{\bar{x}}_{k}}{\mu }\right)$ indicates the impact of each determinant on the desired health outcome, i.e. how much change in the dependent variable is associated with one unit of change in the explanatory variable. The CI indicates the extent of unequal distribution of each determinant across economic groups. The second component, the unexplained portion, is the part of the inequality that cannot be explained by systematic variation in the contributors (determinants) across economic groups.

To decompose, the values of the all of the included variables in Equation (3) should be computed. First, the coefficients (**β_k_**) of the explanatory variables are calculated. To do this, we need to conduct a regression analysis using an appropriate regression model. In the second step, the means of the health variable (**μ**) and each determinant $\left({\bar{X}}_{k}\right)$ are calculated. Thirdly, by multiplying the mean of each determinant by the corresponding coefficients and dividing the result by the mean of the health variable, we can calculate the elasticity of each determinant. In the fourth step, CIs for the health variable (**C**), determinants (**C_k_**) and the generalized CI of the error term (**GC_ε_**) are calculated. **C_k _**can be calculated by Equation (1), but in this case the value of the determinant for **i**th individual and the mean of the determinant are substituted for **y_i _**and **μ **respectively. Now that all the variables in Equation (3) have been calculated, in a fifth step we can reveal the contribution of each determinant to inequality by multiplying the elasticity of each determinant by its concentration index $\left(\frac{\beta_{k}{\bar{x}}_{k}}{\mu }\right)C_{k}$. This is the absolute contribution of each determinant to the measured inequality. Taking the absolute contribution, one can note that the contribution to inequality is the result of two factors: (1) a marginal effect of each determinant on the health variable and (2) the distribution of the determinant based on economic status. In a sixth step, to calculate the percentage contribution, the absolute contribution of each determinant is divided by the CI of the health variable $\left(\frac{\beta_{k}{\bar{x}}_{k}}{\mu }\right)C_{k}/C$.

In our study, mental health is considered as a binary outcome. Thus, to calculate the coefficients of the explanatory variables (**β_k_**), a Logit model (logistic regression) was applied. In the Logit method, the dependent variable is not entered into the model directly, rather it is first transformed into a Logit variable, i.e. a natural logarithm of odds. This makes it possible to conduct a linear decomposition [25]. Hence, taking equation (2), the appropriate regression model for the linear decomposition method will be:

(4) $$\text{Ln}\;\text{odd}{\text{s}}_{\text{mental}\;\text{health}}=\alpha +\sum \beta_{i}x_{i}+\varepsilon_{i}$$

The point that should be highlighted here is that the CI of the dependent variable based on the Logit model shows the degree of inequality in the natural logarithm of the **predicted odds **of mental health. This CI describes the inequality in **predicted **mental health given the observed values of the **x **(determinants) and is different from **overall CI **that can be computed applying equation (3). Therefore, in the CI of dependent variables based on the Logit model attention is focused on the first term of equation (3), i.e. the predicted inequality as measured by ***C_ŷ_***:

(5) $$C_{\hat{y}}=\sum_{k}\left(\frac{\beta_{k}{\bar{x}}_{k}}{\mu }\right)C_{k}$$

In the present study, all analyses were performed in Stata software version 10/SE [26].

## Results

As shown in Table 1, 40% of the study sample comprised men; most participants were employed (61%), and belonged to the age group of 25-44 years (43%); the majority of people were literate (90%), married (86%), and under health insurance coverage (73%).

**Table 1:** Prevalence of mental disorders in terms of determinant variables among Tehran residents aged 15 and above in 2007

Variable	Sample size (%)	Suspected cases (N)	Prevalence rate (%)
**Gender (male)**	8955 (40.4)	2686	30.0

**Gender (female)**	13180 (59.6)	5272	40.5

**Ethnicity (Fars)**	13012 (58.8)	4484	34.4

**Ethnicity (Azeri)**	5645 (25.5)	2295	40.6

**Ethnicity (other)**	3478 (15.7)	1322	38

**Employment status (employed)**	13707 (61.9)	4475	32.6

**Employment status (unemployed)**	8428 (38.1)	3504	41.5

**SES (richest)**	4393 (19.8)	1253	28.5

**SES(second richest)**	4389 (19.8)	1423	32.4

**SES(middle)**	4403 (19.9)	1560	35.4

**SES(second poorest)**	4387 (19.8)	1742	39.7

**SES(poorest)**	4563 (20.6)	2148	47

**Education (academic)**	4769 (21.5)	1308	27.4

**Education (pre-academic)**	15131 (68.3)	5611	37

**Education (illiterate)**	2235 (10)	1107	49.5

**Health insurance (have)**	16249 (73.3)	5707	35.1

**Health insurance (not have)**	5886 (26.7)	2277	38.6

**Marital status (married)**	19140 (86.4)	6631	34.6

**Marital status (widowed)**	2154 (9.7)	1066	49.4

**Marital status (divorced)**	397 (1.7)	193	48.6

**Marital status (single)**	444 (2)	144	32.4

**Age group (15-24)**	5201 (23.5)	1642	31.5

**Age group (25-44)**	9556 (43.1)	3204	33.5

**Age group (45-64)**	4992 (22.5)	1986	39.7

**Age group (≥ 65)**	2386 (10.7)	1197	50.1

**Household size (1)**	983 (4.4)	506	51.4

**Household size (2-5)**	19244 (87)	6790	35.2

**Household size (≥ 6)**	1908 (8.6)	779	40.8

**District 1**	931 (4.2)	272	29.2

**District 2**	934 (4.2)	301	32.2

**District 3**	871 (4)	205	23.5

**District 4**	1542 (7)	588	38.1

**District 5**	1450 (6.5)	445	30.6

**District 6**	917 (4.1)	285	31

**District 7**	919 (4.1)	333	36.2

**District 8**	1052 (4.7)	425	40.4

**District 9**	929 (4.2)	340	36.6

**District 10**	933 (4.2)	377	40.4

**District 11**	951 (4.3)	308	32.3

**District 12**	958 (4.3)	339	35.3

**District 13**	948 (4.3)	378	39.8

**District 14**	1154 (5.2)	440	38.1

**District 15**	1068 (5)	436	40.8

**District 16**	935 (4.2)	413	44.1

**District 17**	937 (4.2)	387	41.3

**District 18**	961 (4.3)	338	35.1

**District 19**	913 (4.1)	333	36.4

**District 20**	960 (4.3)	369	38.4

**District 21**	944 (4.2)	345	36.5

**District 22**	928 (4.2)	333	35.8

Over one third of the investigated population (at the time of study) was detected as likely suffering from a mental disorder (36.4%, range 35.9-36.8). Prevalence of mental disorders in terms of explanatory variables (determinants) is shown in Table 1.

Table 2 shows the adjusted association between mental health and its determinants according to Logit model. We found that some of determinants - including being female, unemployed, living in a household with low socioeconomic status, illiteracy, not having any health insurance coverage and being divorced - increased the odds of mental disorders.

**Table 2:** Adjusted associations between mental health and its determinants based on the Logit model among Tehran residents aged 15 and above in 2007

Variable	Coefficient	P-value	Adjusted odds ratio	95% Confidence interval	
				
				Low	High
**Gender (male)**			1		

**Gender (female)**	.4860	.000	1.62	1.51	1.72

**Ethnicity (Fars)**	-.0898	.029	.91	.84	.98

**Ethnicity (Azeri)**			1		

**Ethnicity (other)**	-.0409	.479	.95	.86	1.06

**Employment status (employed)**			1		

**Employment status (unemployed)**	.2438	.000	1.27	1.18	1.37

**SES (richest)**			1		

**SES(second richest)**	.1204	.019	1.12	1.02	1.24

**SES(middle)**	.1518	.004	1.16	1.05	1.28

**SES(second poorest)**	.2812	.000	1.32	1.19	1.47

**SES(poorest)**	.3889	.000	1.47	1.30	1.66

**Education (academic)**			1		

**Education (pre-academic)**	.2122	.000	1.23	1.13	1.34

**Education (illiterate)**	.3369	.000	1.40	1.21	1.61

**Health insurance (have)**			1		

**Health insurance (not have)**	.1433	.006	1.15	1.07	1.24

**Marital status (married)**	.0066	.916	.99	.77	1.27

**Marital status (widowed)**	.1113	.480	1.11	.86	1.45

**Marital status (divorced)**	.5341	.000	1.70	1.21	2.38

**Marital status (single)**			1		

**Age group (15-24)**	-.5529	.000	.55	.50	.65

**Age group (25-44)**	-.4542	.000	.63	.55	.72

**Age group (45-64)**	-.2277	.000	.79	.70	.90

**Age group (≥ 65)**			1		

**Household size (1)**			1		

**Household size (2-5)**	-.0811	.404	.92	.76	1.11

**Household size (≥ 6)**	.1000	.358	1.10	.88	1.37

**District 1**	.3193	.008	1.37	1.08	1.74

**District 2**	.4763	.000	1.61	1.27	2.03

**District 3**			1		

**District 4**	.7420	.000	2.10	1.69	2.59

**District 5**	.4326	.000	1.54	1.24	1.91

**District 6**	.3240	.007	1.38	1.08	1.75

**District 7**	.5764	.000	1.77	1.40	2.24

**District 8**	.6723	.000	1.95	1.56	2.45

**District 9**	.5047	.000	1.65	1.30	2.10

**District 10**	.6654	.000	1.94	1.53	2.46

**District 11**	.3957	.001	1.48	1.17	1.87

**District 12**	.4398	.000	1.55	1.22	1.96

**District 13**	.7509	.000	2.11	1.67	2.67

**District 14**	.6674	.000	1.94	1.55	2.44

**District 15**	.7024	.000	2.01	1.60	2.54

**District 16**	.8382	.000	2.31	1.82	2.93

**District 17**	.6837	.000	1.98	1.55	2.52

**District 18**	.5179	.000	1.67	1.32	2.13

**District 19**	.5212	.000	1.68	1.32	2.14

**District 20**	.7636	.000	2.14	1.69	2.71

**District 21**	.6456	.000	1.90	1.51	2.40

**District 22**	.6661	.000	1.94	1.54	2.45

**Figure 1:**
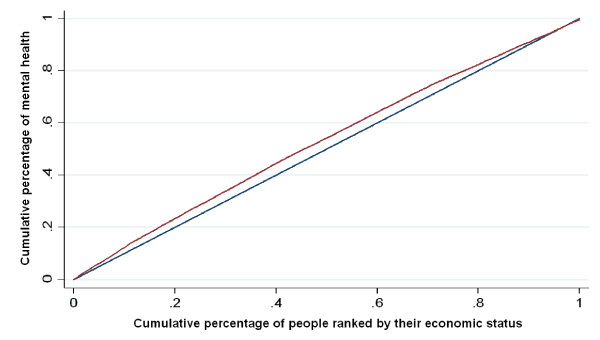
**Concentration curve of mental health in Tehran, 2007**.

Figure 1 illustrates the CC of mental health indicating that people in Tehran who live in households with lower economic status do suffer from higher rates of mental disorders than those with higher economic status. The overall CI of mental health in Tehran was -0.063 (95% CI = -0.070--0.057). The explained component of the overall CI (CI ofLn odds_mental health _= C*_ŷ_*) was -0.051. This component shows that variables entered into the current model were able to explain approximately 80% of the surveyed inequality in mental health in Tehran. The rest of the inequality (approximately 20%) was a residual component of overall CI that had a value of -0.0119. The residual shows the portion of the inequality in mental health that cannot be explained by systematic variation in the variables (entered into the Logit model) across socioeconomic groups; therefore, it cannot be decomposed. In other words, there are other variables or factors that account for this unexplained part of inequality, but the data for those variables were not collected.

The Means and CIs of mental health (Ln odds_mental health_), explanatory variables, absolute contributions and percentage contributions of determinants of mental health inequality are shown in Table 3. In this table, **C_k _**represents the CIs of the explanatory variables. We can see that being female, unemployed, illiterate, widowed or divorced all have negative CIs, meaning that they are more concentrated among people of lower economic status. In contrast, being married or under 65 years old has a positive CI and is more concentrated among those of higher economic status.

**Table 3:** Results for decomposition of concentration index of mental health in Tehran

Variable	Mean	Elasticity	**C**_**k**_^**a**^	Absolute contribution to **C**^**b**^	Percentage contribution to C (%)
**Gender (male)**					
**Gender (female)**	.6047	.0967	-.0057	-.0005	**.9**
**Ethnicity (Fars)**	.5918	-.0175	.0667	-.0011	
**Ethnicity (Azeri)**					
**Ethnicity (other)**	.1513	-.002	-.0807	.0001	**1.9**
**Employment status (employed)**					
**Employment status (unemployed)**	.3819	.0306	-.1114	-.0034	**6.5**
**SES (richest)**					
**SES(second richest)**	.2133	.0084	.3595	.003	
**SES(middle)**	.214	.0107	-.0678	-.0007	
**SES(second poorest)**	.2132	.0197	-.4951	-.0097	
**SES(poorest)**	.1458	.0186	-.8542	-.0158	**44.7**
**Education (academic)**					
**Education (pre-academic)**	.6834	.047	-.0388	-.0018	
**Education (illiterate)**	.101	.0112	-.4714	-.0052	**13.4**
**Health insurance (have)**					
**Health insurance (not have)**	.2658	.0125	-.13	-.0016	**3**
**Marital status (married)**	.8653	.0018	.0435	.000	
**Marital status (widowed)**	.0973	.0035	-.3353	-.0011	
**Marital status (divorced)**	.0171	.0030	-.1063	-.0003	
**Marital status (single)**					**2.5**
**Age group (15-24)**	.2387	-.0434	.1172	-.005	
**Age group (25-44)**	.4387	-.0656	.0235	-.0015	
**Age group (45-64)**	.2291	-.0171	.0154	-.0002	
**Age group (≥ 65)**					**13.1**
**Household size (1)**					
**Household size (2-5)**	.8693	-.0233	.0231	-.0005	
**Household size (≥ 6)**	.0861	.0028	.0577	.0001	**.6**
**District 1**	.042	.0044	.3335	.0014	
**District 2**	.0421	.0066	.3206	.0021	
**District 3**					
**District 4**	.0696	.017	.1041	.0017	
**District 5**	.0655	.0093	.2709	.0025	
**District 6**	.0414	.0044	.2432	.001	
**District 7**	.0415	.0078	.0417	.0003	
**District 8**	.0475	.0105	.0464	.0004	
**District 9**	.0419	.007	-.1328	-.0009	
**District 10**	.0421	.0093	-.2218	-.002	
**District 11**	.0429	.0055	-.1038	-.0005	
**District 12**	.0432	.0062	-.129	-.0007	
**District 13**	.0428	.0105	-.0301	-.0003	
**District 14**	.0521	.0115	-.1194	-.0013	
**District 15**	.0482	.0482	.0482	-.0026	
**District 16**	.0422	.0422	.0422	-.0028	
**District 17**	.0423	.0423	.0423	-.003	
**District 18**	.0434	.0434	.0434	-.0014	
**District 19**	.0412	.0412	.0412	-.0016	
**District 20**	.0433	.0109	-.169	-.0018	
**District 21**	.0426	.0091	.1167	.001	
**District 22**	.0419	.0091	.2132	.0019	**12.5**
**Ln odds of the mental health**	**3.036**		**-.0518**	**-.0518 (total sum)**	
**Residual component**		**-.0119**			

^a ^**C_k _**= concentration index of explanatory variables (determinants)

^b ^**C = **concentration index of mental health

**Figure 2:**
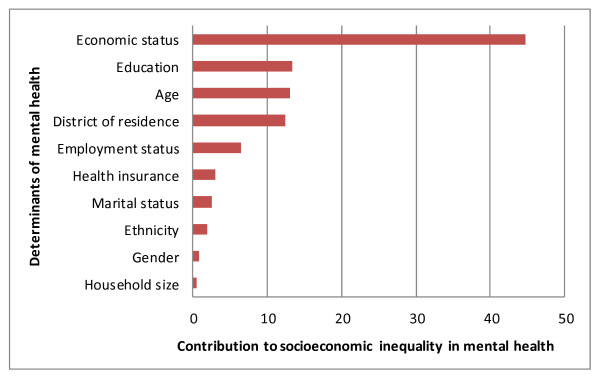
**Contributions of determinants to economic inequality in mental health among Tehran residents aged 15 and over, 2007**.

As Table 3 and Figure 2 show the decomposition analysis revealed that the economic status makes the largest contribution to socioeconomic inequality in mental health (44.7%). Education status (13.4%), age group (13.1%), district of residence (12.5%) and employment status (6.5%) follow in respective importance. To calculate the contributions of variables with several categories (such as economic status, district of residence etc.) the contributions of categories (dummies) in each variable were combined. As this table shows, the CI of Ln odds_mental health _(i.e. -0.051) can be phrased as the **total sum **of the absolute contributions of the explanatory variables.

It is worth noting that no significant differences were seen when inequality was measured and decomposed for men and women separately.

## Discussion

To the best of our knowledge, studies on the decomposition of socioeconomic inequality in mental health have so far been restricted to developed countries, meaning that we lack such analyses in developing countries. In the current study, for the first time, we have attempted to explain the socioeconomic inequality in mental health in Iran's capital, Tehran, by adopting a CI decomposition approach. This can help us to get to root causes of socioeconomic inequalities in mental health in our societies, something which is vital for policy purposes. The mental health CI (of Ln odds_mental health_) in our study revealed that mental health is unequally distributed among Tehran's residents. Indeed, the negative value of this CI shows, as a number of other studies in the other parts of the world (particularly in developed countries) did [27-31], that mental disorders are disproportionately concentrated among people of lower socioeconomic status. In addition, decomposition of mental health inequality revealed that all of the explanatory variables have positive contributions to socioeconomic inequality in mental health. A positive contribution means that the combined effect of the marginal effect of the desired determinant and its distribution based on economic status increases socioeconomic inequality in mental health. This can occur because either the desired determinant is more prevalent among people of lower economic status (negative C_k_) and is associated with a higher risk of mental disorders, or because the determinant is more prevalent among those of higher economic status (positive C_k_) and is associated with a lower risk of mental disorders. Further, in the present study economic status accounted for most of the existing socioeconomic inequality in the mental health of Tehran's residents. In fact, this variable alone is responsible for 44% of the economic inequality in mental health. The next explanatory variables with relatively large positive contributions to socioeconomic inequality in mental health are respectively education, age, district of residence and unemployment. Gender, ethnicity, health insurance coverage and marital status also make a relatively minor positive contribution to inequality. Unemployment, being without health insurance and being female are more prevalent among the low economic quintiles and are associated with a higher risk of mental disorders.

The decomposition method helps to quantify the contributions of determinants to socioeconomic inequality in health [25,32]. Thus, while in this study economic status per se accounts for 44.7% of wealth-related inequality in mental health, 55.3% is due to the fact that important determinants of mental health such as illiteracy or unemployment are strongly correlated with economic status. Thus, decomposition is important as it combines monitoring of inequality and understanding its determinants [25,32].

It should be noted that no weight was used in analysis to adjust for the rate of non-response. This might affect the results in some ways: the non-response cases might be mostly from people who suffer from mental disorders and consequently the prevalence rate could be underestimated; or the measured inequality could be higher, if most of the non-response cases were from people with higher socioeconomic status and did not have mental disorders. However, as the non-response rate was low, the authors decided not to incorporate it in the analyses.

When comparing the results of this study with other studies, we should be aware of differences in the way of quantifying economic status and measuring mental health. When measuring economic status, one method is to use "direct" measures, such as income, expenditure or consumption: however, both income and consumption data are expensive and difficult to collect and consequently might result in bias. These concerns have prompted researchers to use data on household assets and other characteristics to create alternative measures of welfare and living standards, termed "proxy" measures. One common method of this type is to use PCA to construct an index of "wealth" from information on household ownership of durable goods and housing characteristics [21,22]. In Tehran's Urban HEART survey, there was no data regarding income, but the data for family expenditures and household assets were available. For the purpose of this study, the authors preferred to use an index based on household assets in order to avoid any potential bias.

It appears that only two previous studies have tried to address the issue of mental health inequality in a manner similar to the present study. Wildman attempted to analyze the causes of mental health inequality over time in the UK in 1992 and 1998 [27]. He showed that there was a decreasing inequality that disfavored the poor during the studied period, and that subjective financial status, next to age, was the major contributor to the inequality of mental health in the UK. Another study was conducted by Costa-Font and Gil in Spain [28], which revealed significant income-related inequality in depression to the disfavor of the poor and that income had the largest contribution to that inequality.

In other relatively similar studies, only the magnitude of inequality in mental health was measured and no decomposition of CI was attempted; in two different studies, Mangalore and colleagues attempted to measure mental health inequality in the UK public [29] as well as among UK's ethnic groups [30]. Both studies revealed that there was inequality that disfavored the poor and minority ethnic groups. Another study in South Korea revealed an increasing pattern of inequality in depression and suicidal behavior in a period of 10 years (1998-2007) [31].

However, although it is difficult to make a straight comparison, it seems that the present findings are in line with international literature--mental health is distributed unequally in societies and economic status accounts for a remarkable share of this distribution.

Decomposition of CI approach is also of particular use for policy purposes, helping us to reveal main entry points for intervention to reduce socioeconomic inequality in health. For the present study, as shown in Table 3, the main entry points and some possible interventions are as follows:

Regarding demographics (age, gender and ethnicity), age had a considerable positive contribution to inequality (Table 3). The CIs (C_k_) of the age groups in Table 3 show that younger age groups are more concentrated at the higher end of economic scale, implying that older age groups (especially those 65 years old and over who are used as the reference group in our study) occupy the lower end of economic scale. To decrease the positive contribution of age to mental health inequality, policy makers should adopt social welfare programmes that lessen the slope of inequality in the distribution of age groups in favor of the elderly. Policies should help elderly people attain or hold higher economic positions in Tehran. Higher levels of financial support for people over 65 years of age in national targeted subsidies plan could be a possible option in this regard.

According to national annual studies of urban household budgets [33], households in Tehran have the highest gross income and expenditure in the country. Nevertheless, there are great socioeconomic differences among households in various districts of the capital city. Southern districts (12, 16, 17 and 19) are more likely to be characterized by illiteracy, unemployment, female-headed and single-headed households, inadequate housing and immigration [34]. Indeed, as the CIs show (Table 3), these districts are home to higher proportions of poorer people than other districts. In contrast, northern districts (1, 2, 3 and 6) are home to households ranking first in social welfare [34]. This very considerable heterogeneity in distribution of households across districts of Tehran (according to the districts CIs in Table 3) could be an explanation for relatively high contribution of "district of residence" to socioeconomic inequality in mental health. Certainly, better urban planning is required to change the current situation. Preventing urban marginalization would be an appropriate strategy. Fortunately, urban marginalization has been prioritized in the Iranian ministry of health's plan to tackle social determinants of health. Tehran's Urban HEART itself is a user-friendly and policy-driven effort to identify and tackle urban characteristics that worsen health [11], although as yet little has been done regarding the response part of tool in the city.

Since our study, along with some other studies [18,35] has revealed the paramount importance of economic status and economic inequality in the unequal distribution of health among Iranian people, bringing health to forefront of every economic and developmental agenda seems logical and urgent. Indeed, we require appropriate strategies to tackle existent economic inequalities and reach the poor in society. Such strategies would then lead to a reduction in health inequalities. Possible relevant strategies could include: (1) identifying vulnerable and disadvantaged groups in terms of demographic and socioeconomic characteristics; (2) directing development to these identified poorest districts; (3) providing extra monetary transfers in the form of income or benefits for the most disadvantaged; (4) consolidating taxation policies to redistribute income from the rich to the poor; and (5) spreading the net of social security to poorer members of society. An important and noteworthy point is that Iran's policy directions, in all areas, lack a clear and applicable definition of equity. The first step in tackling socioeconomic inequalities and thus health inequalities would be in our view to clarify the concept of equity as suggested here. The targeted subsidies plan [36], Iran's health innovation and science development plan by 2025 [37] and the national social safety net programme are the main programmes that attempt to fulfill mentioned strategies, although studies are still required to assess their effectiveness.

Although from an educational equity point of view, as a result of a number of initiatives such as a literacy campaign, adult literacy rates in Tehran have improved significantly over the past twenty years [34,38], the contribution of education to socioeconomic inequality in mental health in Tehran suggests that, to establish health equity, education should receive greater focus and investment. This applies especially to women, who still have higher rates of illiteracy (11.1%) compared to men (6.5%) in Tehran [34]. Expanding the "home schooling" plan of Iran Literacy Organization in Tehran could be an appropriate strategy in this regard.

According to the most recent data, Tehran has one of the highest rates of unemployment (14.2%) in Iran [39]. Considering the detrimental effects of unemployment on mental health [40-43] and the concentration of the unemployed at the lower end of the economic scale in Tehran (as shown in the CI for unemployment in Table 3), the considerable positive contribution of unemployment to inequality in mental health is understandable. To reduce this contribution, providing decent and secure employment, especially for the increasing number of well-educated people, seems urgent. Some of the main strategies in this regard, as outlined in the country's fifth development plan, are (1) directing the majority of subsidies to production rather than consumption; (2) supporting and financing informal employment, especially small home businesses and community-based initiatives; (3) correcting the imbalance in the distribution of opportunities across districts; and (4) placing special emphasis on women in employment agendas.

Ethnicity, health insurance and marital status were the variables with relatively minor positive contributions to inequality. By establishing the strategies mentioned above to tackle socioeconomic inequalities and reach the poor and less-advantaged, the positive contribution of these variables, which mainly derives from their unjust concentration at the lower end of the economic scale, could in our view be redressed.

A matter that needs more contemplation and has strong implications for mentioned policy is that the association between low socioeconomic status and mental disorders does not appear to be a one-way relationship; it is a vicious cycle, resulting from a dynamic interrelationship between the two variables [2]. More importantly, this vicious cycle is established with differential and inequitable effects across socioeconomic groups, that is, with greater adverse effects on the poor [2]. Thus, along with measures for social and economic improvements and expansion of social welfare nets across society, there should be policies and measures that target the mentally ill and prevent the discrimination and socioeconomic deprivation associated with mental disorders. It is important to create policies that provide treatment and care--with lower out-of-pocket expenditure--for mentally ill people, or introduce sickness benefits for mental disorders in the workplace.

Apart from national implications, the findings and policy implications of the present study can also be contextualized in the international and, particularly, the regional levels. From socioeconomic and demographic perspectives, Middle East and North Africa (MENA) countries share some characteristics that put them in similar circumstances in terms of mental health and mental health equity. In brief, these characteristics are a high youth population (40% of total population), increasing elderly population, high unemployment, fast and awkward urbanization, changes in family structure, unplanned transition to modernity, a growing rate of educated but poor people, political instability and social disturbances [44]. These socioeconomic characteristics have resulted in commonalities in the mental health status of MENA countries. Currently, mental health is more threatened, and mental disorders take a greater toll on societies, than at any time in the past, and there has been a steady growth in the prevalence of mental disorders [45-47]. Of 24 MENA countries, approximately all have adopted a community mental health approach and developed a national mental health program during the past two decades [48]. However, as with Iran, no mental health inequality-directed program has been under consideration yet, nor has any study been conducted to assess the mental health equity impacts of the current programs. Policies are currently only directed to lower the prevalence rate of mental disorders. However, it is hoped that the findings of this study can encourage MENA countries to investigate and explain the socioeconomic inequality in the distribution of mental health; similarly, they can take into consideration the mental health impacts when developing socioeconomic policies. Further, the present study can show countries that it would be more effective to reassess and redesign their current national programs by taking into consideration both the distribution and prevalence of mental disorders.

### Limitations

As mentioned previously, as compared to other studies that used household income to measure economic status, this study has the advantage of measuring economic status using a more accurate method called PCA. This method has fewer limitations compared to direct measure in developing countries [14,21]. In addition, instead of applying linear regression to decompose inequality in a non-linear setting, the current paper used a more appropriate method to accomplish its goals. Nevertheless, our study has its own limitations.

Since our data were drawn from a cross-sectional study, attribution of causal interpretations to the results should be done with caution. In fact, longitudinal data are necessary here. Further, because our study only covers subjects aged 15 years and over, we have no information on the distribution of mental health in children and juveniles, who make up a very significant proportion of the Iranian population. Therefore, surveys that cover all population groups could better represent the socioeconomic inequality in mental health and the resulting ratified policies could better tackle the socioeconomic determinants of mental health inequality. The other limitation of our study is the higher proportion of women in the analysis. Our intention was to have approximately equal numbers of women and men in the study, in accordance with Tehran's population structure; however, when the GHQ-28 was administered, the available member of the household was interviewed, who in most cases was a woman. This could affect the results in a number of ways. Indeed, some of the socioeconomic inequality in mental health could be due to this issue. This should be borne in mind when interpreting our findings.

## Conclusions

In conclusion, we can state that scaling up social and economic policies that are in alignment with health policies could bridge the current avoidable, unjust and unethical gap between the mental health of advantaged and disadvantaged groups in Tehran - a gap that arises from inequalities in the social determinants of mental health. This means that Intersectoral collaboration between the health system and other social and economic authorities is urgent to reduce the socioeconomic inequality in mental health in the capital city.

## Competing interests

The authors declare that they have no competing interests. Also, the views expressed in this paper are those of the author(s) and do not necessarily represent the views or policies of the World Health Organization.

## Authors' contributions

EKM was the main investigator, and wrote the manuscript draft. ASF made substantial contribution to analysis and interpretation of data and helped in writing the paper. RM made substantial contribution to analysis and interpretation of data. MAL made substantial contribution to acquisition of data and analysis and interpretation of data. AAN had a critical advisory role in preparation and refinement of draft and manuscript. ARH made critical comments that helped in the interpretation of results and final writing of the paper. All authors read and approved the final manuscript.
